# Agreement and Reliability of Parental Reports and Direct Screening of Developmental Outcomes in Toddlers at Risk

**DOI:** 10.3389/fpsyg.2021.725146

**Published:** 2021-09-28

**Authors:** Juan Giraldo-Huertas, Graham Schafer

**Affiliations:** ^1^Department of Psychology of Development and Education, Universidad de la Sabana, Chía, Colombia; ^2^The School of Psychology and Clinical Language Sciences, University of Reading, Reading, United Kingdom

**Keywords:** parental reports, developmental screening, children at risk, reliability and agreement studies, low-middle income countries, receiver operating characteristic (ROC) analysis

## Abstract

Developmental screening is a practice that directly benefits vulnerable and low-income families and children when it is regular and frequently applied. A developmental screening tool administered by parents called CARE is tested. CARE contains a compilation of activities to report and enhance development at home. Hundred and fifty-seven families in Bogotá (Colombia) initially responded to a call to participate in developmental screening tools’ validation and reliability study. All children (Average: 42.7 months old; *SD*: 9.4; Min: 24, Max: 58) were screened directly by trained applicants using a Spanish version of the Denver Developmental Screening test [i.e., the Haizea-Llevant (HLL) screening table]. After a first screening, 61 dyads were positive for follow-up and received a second HLL screening. Fifty-two out of 61 dyads use and returned CARE booklet after 1-month screening at home. The comparative analysis for parent reports using CARE and direct screening observation included (a) the effects of demographic variables on overall and agreement, (b) agreement and congruence between the CARE report classification and direct screening classification (“At risk” or “Not at risk”), (c) receiver operating characteristic analysis, (d) item-Level agreement for specific developmental domains, and (e) acceptability and feasibility analysis. Results and conclusions show the parental report using the CARE booklet as a reliable screening tool that has the potential to activate alerts for an early cognitive delay that reassure clinicians and families to further specialized and controlled developmental evaluations and act as a screen for the presence of such delay in four developmental dimensions.

## Introduction

Attention to screening tools in low-and-middle income countries (ongoing: LMIC) settings has grown recently (Boggs et al., 2019). However, only population-level tools (i.e., instruments for monitoring countries or regional status) have been shown to have acceptable accuracy, reliability, and feasibility for routine use in health and educational systems. Individual-level tools (i.e., instruments to measure cases or single participant assessment) are not frequently reported to have utility in planning for direct early interventions. Efforts for optimal monitoring and screening tools have a direct relationship with the Nurturing Care Framework (Britto et al., 2017; WHO, 2020). The Nurturing Care Framework has inspired a considerable literature for early interventions in LMIC (Trude et al., 2021). Reviews of previous screening and surveillance projects around parenting effects on children development, shown how high nurturing interventions reduce negative effects of scarce and adverse environments (Lu et al., 2020; Tann et al., 2021). However, there is no complete or permanent program in an LMIC that ensures constant and relevant evidence-based approaches to monitoring and assessment of child development or nurturing status (Milner et al., 2019). Along with monitoring, even in high income countries, indicators and information to design interventions and programs guided by developmental screening (DS) to reduce social and educational inequity are incomplete (NASEM, 2019). The NASEM report showed how, before the COVID-19 pandemic, standard health information systems needed improvements in research and data sources, to fill important gaps in knowledge about child intervention programs to identify promising program features to implement effectively at scale. The same efforts are needed in getting accurate information including a call for action through developmental monitoring and screening in LMIC (Goldfeld and Yousafzai, 2018). Increasing developmental monitoring and screening of children’s outcomes can optimize early intervention referrals, assessments, and eligibility (Barger et al., 2018). Also, in LMIC like Colombia, where this pilot study take place, screening tools for children monitoring about developmental risks should fight against the impact of social inequalities in children’s development, a primary socio-political goal and where testing children directly by public administration services it is not always accessible in vulnerable populations (Rubio-Codina and Grantham-McGregor, 2020).

The main aim in the present study is related to the Compilation of Activities to Report and Enhance development (Ongoing: CARE), a booklet created to obtain screening information of daily activities of interaction between parents or caregivers with children in vulnerable families living in Colombia. The consequent aims of the current study are threefold:

(1) Explore the diagnostic characteristics and performance of CARE as a tool for DS using parent reports, with item agreement analysis at the individual level between parent reports and direct assessment in particular domains, as set out above.
(2) Examine consistency between parental reports using CARE and classification and scores using an external screening in the domains of personal-social skills, language and logico-mathematical reasoning, fine motor-adaptive and gross motor skills. We expect to find similar results to prior research showing good agreement between parent report and direct testing of social, language and gross motor skills, but somewhat weaker agreement in fine motor skills (Miller et al., 2017).
(3) Obtain relevant data to identify the validity of CARE, with feedback of the findings to both academic and institutional administrators engaged in participant enrollment.

Following paragraphs extend the rationale for every specific aim.

The first aim explores the diagnostic characteristics and performance of a new DS tool administrated by parents and compared with an external screening tool measurement. Improving screening and developmental status measurement in early child development is feasible, but several coverage and quality characteristics remain unreachable for evidence-based interventions in LMIC (Milner et al., 2019). Interventions with simpler, routinary and including multi-domain outcome measurement needs well-designed tools. DS tools reduce financial and time costs for fundamental research and public health activities, such as assessing early developmental status at an individual level (Johnson et al., 2008), even in LMIC (Tann et al., 2021). However, several decision-making steps are required when DS tools are included in interventions, monitoring programs, assessments, or research (Nadeem et al., 2016). In the last decade, different studies have evaluated DS tools deployed at primary healthcare services in LMIC (Fischer et al., 2014; Fernald et al., 2017; Boggs et al., 2019). These three studies rated 14 individual-level tests, applying common criteria for validity, reliability, accessibility of application, required training, administration time, cultural adaptability, geographical uptake, and clinical relevance and utility. Utility was only considered for the category of individual-level measurement tools. Boggs et al. (2019) excluded the costs of the tool (i.e., the budget necessary to buy and use the materials and to train personnel) from the criteria listed by Fischer et al. (2014). The review of 14 individual-level tests indicated higher ratings of administration time or reliability compared with population-level and ability-level tools (Boggs et al., 2019). Of these 14 individual-level tests, 36% (*n* = 5) had a higher rating for both administration time and reliability: namely, the Ages and Stages Questionnaire (ASQ), the Denver Developmental Screening Test (DDST), the Guide for Monitoring Child Development (GMCD), the ICMR Psychosocial Development Screening Test, and the Parents’ Evaluation of Developmental Status (PEDS). Those review studies did not find any screening tool that was particularly used or designed in Colombia (Fischer et al., 2014; Boggs et al., 2019).

The Colombia’s Ministry of Health uses the Abbreviated Development Scale (Ongoing ADS; in Spanish, *Escala Abreviada del Desarrollo*; Ortiz, 1991) not like a screening tool, but in different institutional scenarios, including children’s centers and public kindergartens around the country, to obtain information about children emotional, cognitive and health conditions. Colombia’s Ministry of Health (Ministerio de Salud de la República de Colombia, 2016) presents the ADS with no published report on its conceptualization, pilot testing, or complete analysis of validity and reliability. A partial validation analysis of the ADS-1 for the language and hearing domain in 4- to 5-year-old children indicated low predictive ability (Sensitivity: 54%, Specificity: 42%) and poor agreement with a gold standard for early detection of language and hearing disorders (i.e., *the Reynell norm-referenced test*) on measuring expressive and receptive language skills, and with tone audiometry and otoacoustic emissions on assessing hearing (Muñoz Caicedo et al., 2013). We can therefore conclude that to the best of our knowledge, it is not a well-designed tool for the Colombian context, following the standards of Boggs et al. (2019). Moreover, the aforementioned rating exercises report the use of a “developmental domain” approach to the relevant screening tools, but not an analysis of “administration of test,” which is recommended by different authors (Fernald et al., 2017; Boggs et al., 2019). The “administration of test” view implies comparing caregiver reports with direct child observation. Vitrikas et al. (2017) described both a parent-completed DS tool as an instrument for obtaining screening information through parent participation, and (as a separate instrument) a directly administered DS tool when information is based on direct observation of the child by a physician or other expert.

The second of the three aims examine the consistency between parental reports using CARE and classification and scores using an external administrated tool, including reliability and agreement analysis. DS still has some unique challenges associated with obtaining accurate data in early childhood, especially in LMIC and families in poverty conditions (Lu et al., 2020). The Early Childhood Development Index (ECDI), for example, is a 10-question survey used in the Nurturing Care Framework to determine whether children are on track in their cognitive and social-emotional development (Richter et al., 2017, 2020). For global, national, and regional level, ECDI information is fundamental, but high-quality and comparable data for individual developmental status is not fully captured by developmental surveys or questionnaires (McCoy et al., 2016, 2018; Lu et al., 2020). Parental reports are a high-quality, reliable alternative to obtaining individual child information via home visits. We define ‘parent report’ in this study as information obtained from a parent using CARE^®^. The CARE is a booklet created to obtain information of daily activities of interaction between parents or caregivers with children, derived from an instrument applied by training specialized personal in a 3-year research program, with a sample of 1173 children under 6 years old and their caregivers in two large territorial regions of Colombia (Cundinamarca and Boyacá), in urban and rural settings (Giraldo-Huertas et al., 2017). The main content of CARE includes activities to report developmental milestones in four domains mentioned before, for two age groups: 24–35 months old and 36–59 months old. Every item in CARE is closely related to one item in the Haizea-Llevant (HLL) Table (Iceta and Yoldi, 2002). The HLL screening table is a DS tool derived from the Denver Developmental Screening Test (DDST) and the Denver Pre-screening Developmental Questionnaire (Frankenburg et al., 1976; Frankenburg, 1987). HLL was selected because the DDST is broadly used and standardized in different countries (Lipkin and Gwynn, 2007; Guevara et al., 2013; Dawson and Camp, 2014), including populated regions in Brazil (Lopez-Boo et al., 2020) and Colombia (Rubio-Codina and Grantham-McGregor, 2020). The HLL is a similar Spanish language version of the DDST, used previously in a long-term health screening program in the Basque Country (Fuentes-Biggi et al., 1992; Rivas et al., 2010). The HLL items included in CARE and the whole designing process follow the components recommended by Nadeem et al. (2016) for construction and validation of assessment tools. Conceptualization and consolidation phases were realized in the IPV (Inicio Parejo de la Vida, “Equal Start in Life”), a research program with previously take place in Colombia (Giraldo-Huertas et al., 2017).

Compare parental reporting and direct assessment are defined as the two main methods used to evaluate child development (Miller et al., 2017). Miller et al. (2017) remark on the need to determine reliability and agreement in parental reports in the early detection of developmental delays, comparing these with direct assessments as a quality control procedure. In a framework for optimal quality in early childhood assessments, reliability and agreement (R&xsA) studies are often expected (Vanbelle, 2017). R&A studies provide information about the quality of measurements, specifically about the ability of a scale to differentiate between the items, despite the presence of measurement error (reliability); and also, about the degree of closeness between two assessments made on the same items (agreement). Good levels of R&A are essential for new measurement tools if they are to be included in clinical decision making and subsequent interventions (Vanbelle, 2017). R&A application may relieve technical concerns about the accuracy of parental reporting (Bennetts et al., 2016; Miller et al., 2017). Parents are an important source of information regarding child skill deficits and atypical behaviors, because they are uniquely positioned to observe and interact with children across various daily interactions at home (Jeong et al., 2019). Also, for developmental monitoring (i.e., healthcare professionals’ practices to make informed clinical judgments about children’s developmental progress based on their own criteria) parent reports might be included to help identify children at risk (Barger et al., 2018; Gellasch, 2019). Developmental monitoring practices with parent reports for individual developmental status and later diagnostic testing may be shorter to administer, thereby reducing costs and increasing developmental delay identification in the regular health visits at 9, 18, and 24–30 months (Miller et al., 2017; Vitrikas et al., 2017; Gellasch, 2019).

Finally, a third aim is to obtain relevant data to identify the validity of CARE in protocols for feedback of the use and individual results to both academic and institutional administrators engaged in participant enrollment. Unfortunately, even in high-income countries, only a small proportion of children regularly receive developmental monitoring in health systems, preventing the detection of early delays and subsequent interventions (Barger et al., 2018). The COVID-19 pandemic may have exacerbated adversity and imposed still more barriers to the optimization of developmental monitoring (Richter et al., 2020; Trude et al., 2021), making parental reports valuable tools for identifying individual children’s developmental status. The present study aims to evaluate consistency between two sources of information—direct assessment and parent report—when classifying at-risk children and measuring child development in four domains (personal-social, language and logico-mathematical reasoning, fine motor-adaptive, and gross motor skills) within a reliability and agreement analysis, and finally, a validity report for inclusion in future institutional or community scenarios.

It is important to note that the parental administration method does not profess to replace any clinical or scientific intervention and will presumably run in parallel with other previously existing or subsequently developed screening and intervention methods for health and educational systems. Specifically, this study review CARE characteristics and initial scopes as a screening tool, and it is not possible to currently considerer that should be used for intervention.

## Materials and Methods

### Participants

**FIGURE 1:**
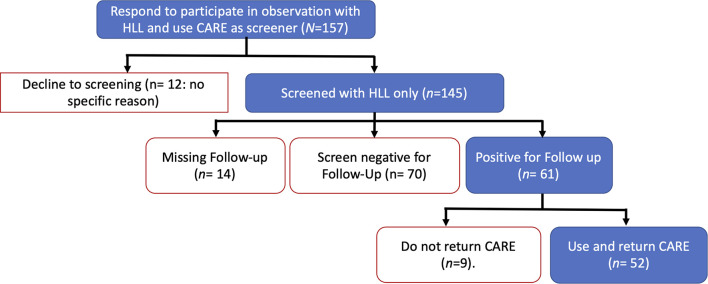
Consort diagram for participants called for screened with the Haizea-Llevant screening table and to use CARE at home. HLL, Haizea-Llevant; CARE, The Compilation of Activities to Report and Enhance development booklet. One-month pass between the positive Follow-up and the caregivers return of CARE booklet used as screening tool.

Participants were dyads of toddlers and principal caregivers recruited at a children’s center pertaining to a community-level social support intervention that was part of a wider government-funded nutritional program. The study’s catchment area included an urban population vulnerable to poverty in the north-west of Bogotá, Colombia. One hundred and fifty-seven families (*N* = 157) initially responded to a call to participate in a study of tools for a future cognitive intervention and completed documentation for informed consent (Figure 1). All children were screened using the HLL screening table (Iceta and Yoldi, 2002). Due to reported application practices for early DS (Alcantud et al., 2015), HLL was applied twice. The first application intent to diminish possible anxiety or fear around working with a health professional in screening settings (Villagomez et al., 2019) and follows the recommended application twice before screening decisions with participants in systems for early detection of developmental disorders (Alcantud et al., 2015). One week later after a first screening with HLL, 61 dyads (85.2%) were positive for follow-up and received a second HLL screening. Some 52 caregivers out of these 61 dyads returned the CARE booklet after using it as a screening tool at home.

The sample included all families who satisfied the following criteria: (1) They had at least one pre-school child (aged 59 months or younger); (2) they were currently in a couple, unless it was unfeasible to talk with one partner (excluding, e.g., partners who traveled a lot, widows, divorcees; (3) they understood written or spoken Spanish; and (4) they were willing to receive a CARE booklet and use it as a screening tool, to the best of their capabilities. Sociodemographic characteristics of the final participants sample are described in Table 1. The procedure to obtain sociodemographic information, described below, does not establish any statistical difference in the profile of families who dropped out of the study at different stages.

**TABLE 1:** Characteristics of the sample for validation of CARE^®^ (*n* = 52).

*Sex of the child*	*n (%)*
Female	23 (44.2)
Male	29 (55.8)
***Age group***	
24–35 months old	9 (17.3)
36–47 months old	25 (48.1)
48–59 months old	18 (34.6)
***Principal caregiver (PC)***	
Mother	29 (55.8)
Relative at home	9 (17.3)
Relative out of home	5 (9.6)
Non-relative at home	2 (3.8)
Non-relative out of home	1 (1.9)
No answer	6 (11.5)
***PC educational level***	
No school experience	1 (1.9)
Incomplete elementary	6 (11.5)
Elementary	5 (9.6)
Incomplete high school	2 (3.8)
High school	18 (34.6)
Technician	9 (17.3)
Incomplete undergraduate	1 (1.9)
Undergraduate	3 (5.8)
Postgraduate	1 (1.9)
No answer	6 (11.5)
***Maternal Employment***	
Employed	34 (65.4)
Unemployed	12 (23.1)
No answer	6 (11.5)
***Type of settlement***	
Urban	39 (75.0)
Non-urban	4 (7.7)
No answer	9 (17.3)
***Socioeconomic national scale^+^***	
Level 1 Very low: Between 1488 and 1606 US Dollar by year or less.	13 (25.0)
Level 2 Low: More than 1606 US Dollar by year but less than one national minimum wage (3.751 USD per year).	19 (36.5)
Level 3 Medium low^++^: less or more than one or two national minimum wage as household income.	14 (27.0)
No answer	6 (11.5)

*^+^Income are exchanged to US dollars in July/2020; ^++^ Sources: MESEP-DNP (2011) and Sánchez-Torres (2015).*

### Measures

Each dyad was interviewed and received:

(1) Sociodemographic information survey (The Questionnaire for Parents and Caregivers General Data; Profamilia, 2010; Giraldo-Huertas et al., 2017).
(2) The Haizea-Llevant screening table (Iceta and Yoldi, 2002).
(3) The CARE booklet.

#### The Questionnaire for Parents and Caregivers General Data

The Questionnaire for Parents and Caregivers General Data (GDQ) was used in the IPV (Inicio Parejo de la Vida—Equal Start in Life) program (Giraldo-Huertas et al., 2017) and contains the 14 variables associated with the socio-cognitive development of children of under 6 years of age in the geographic region of interest, including items from the ENDS (Encuesta Nacional de Demografía y Salud—Colombian National Survey of Demographics and Health; Profamilia, 2010). The GDQ comprises 68 questions in eight modules that obtain data about the social, demographic and health characteristics of children under 6 years-old and their families. All questions were answered by the mother or primary caregiver of each child. The survey took approximately half an hour per participant.

#### The Haizea-Llevant Screening Table

**FIGURE 2:**
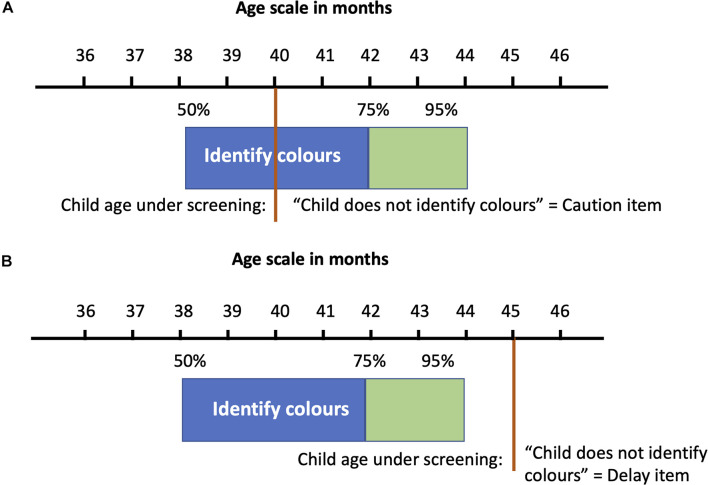
**(A,B)** Examples of Caution and Delay answers in “Identify colors” item in Haizea-Llevant.

The HLL (Fuentes-Biggi et al., 1992; Iceta and Yoldi, 2002; Rivas et al., 2010) was used by the research team for individual assessment of children. The individual developmental performance score is defined as the number of age-appropriate test items of a domain in HLL that a child can successfully pass or not. For nominal classification, a “Caution” is recorded when an age-appropriate item is not passed. If the child is older than the limit age for the 95% of the standardization population passing the item, and does not pass it, that item is recorded as a “Delay.” As example for an item (“Identify colors”) in the domain of language and logic-mathematical reasoning: if a child is 40 months old and does not identify colors when these are pointed out by the interviewer, this is interpreted as a “Caution” item (Figure 2A); if a child is over 44 months old and does not identify colors during the observation with the HLL, this is interpreted as “Delay” item (Figure 2B).

The counting of Caution and Delay items enables scoring of the overall test and helps the interpretation of the screening, permitting additional evaluations and referrals as appropriate (Vitrikas et al., 2017). For nominal classification of the results, if the child at least one Delay item or at least two Cautions, he/she would be classified “At risk.” No Delay answers and just one Caution answer would lead to a classification of “Passing.” Henceforth, we classify those participants “Passing” the HLL as “Not at risk.” For developmental domain analysis, values were scored following a recent approach for the Denver II test, using an analysis of the distribution of items in the Haizea-Llevant tool according to age (Drachler et al., 2007; Lopez-Boo et al., 2020). A quantitative coefficient for continuous variable analysis in the Haizea-Llevant tool was obtained by scoring the Delayed items as minus one point (–1) and Caution items as zero (0) and totaling the result. A Positive answer or performance in HLL is scored with one point if child’s performance is equal to or better than that of 50% or more of the standardization population for their age.

#### The CARE Booklet

**FIGURE 3:**
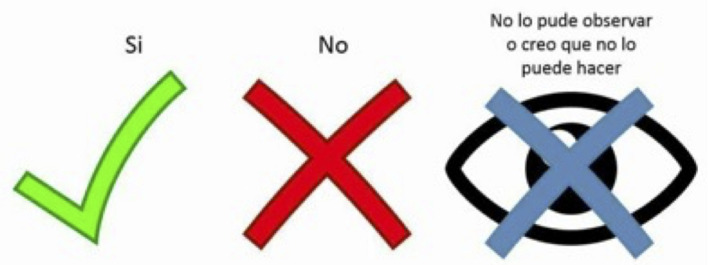
Report icons of parent–child interaction in CARE booklet.

Parents, mainly mothers to our case (55.8%), received a CARE booklet to be used as a screening report. The report consists of a mark over an icon (Figure 3), for which the parent or caregiver chooses Sí (“Yes”) if the skill or behavior was observed in interaction with the child, No if the skill or behavior was not observed in interaction with the child, or *No lo pude observar o creo que no lo puede hacer* (“I couldn’t observe it or I believe they can’t do it”) if the parent did not have an opportunity to observe if the skill or behavior were attainable by the child. The two options fall under the same question because the main intention with the booklet is the report of interactions, not recalls or beliefs about the children’s skills. The components of the CARE booklet keep the same dimensions but vary in the complexity of items between 24–35 months old and 36–47 months old. The content for 36–47-month-old children is the same as for 48–59-month-olds. The CARE instrument has 47 items in four domains comparable with the HLL observations: (a) personal-social (11 items), (b) language and logico-mathematical reasoning (20 items), (c) fine motor-adaptive (9 items), and (d) gross motor (7 items). It also includes an exploration of socio-cognitive development in context, in the use of Core Knowledge Systems (Kinzler and Spelke, 2007; Callaghan et al., 2011). The “Core Knowledge” components inquired with CARE are related to spontaneous and autonomous play, counting, geospatial orientation, age-pair interactions and outdoors activities. The Core Knowledge components used do not differ between each age-group booklet. The nominal classification and agreement analyses do not include the Core Knowledge components.

For nominal classification with the results in CARE, we followed the HLL scoring system, but included an arbitrary range for the not reported interactions when parents use the “I can’t observe it or I believe he/she can’t do it” option: if the child at least one Delay or at least two Cautions or at least four unanswered items (i.e., “I can’t observe it or I believe he/she can’t do it”) he/she was classified “At risk.” ‘No Delay’ answers or less than two Cautions or ≤3 not answered items he/she would be classified ‘Not at risk.’ A quantitative coefficient for continuous variable analysis in CARE performance was obtained by scoring the Delayed items with –1 and Caution items with 0. A positive answer or performance in CARE was scored with 1 point.

### Procedure

Children who screened positive for risk in a first screening, participated at a follow-up HLL screening at children’s centers (CCs). The follow-up was performed by three trained assessors in an individual meeting with caregivers and children. During the second and final HLL screening, one of the assessors applied a survey to obtain sociodemographic information. Survey and screening application lasted less than 30 min. For children who screened positive in the initial session, a member of the research team contacted caregivers in the CC to administer the follow-up screen using HLL. A licensed psychologist then checked that assessors had completed all evaluations and proceeded to deliver a copy of the CARE booklet. Parents watched an instructional 2-min video on how to report children’s activities using the CARE booklet. Families were instructed and directed explicitly to principal caregivers (Table 1) to carry out the activities and return the booklet as soon as possible but not less than 1 month after receiving it. After they had watched the video with the reporting instructions, the CARE booklet was delivered to the caregiver with the following items in a toy bag for each child: five wooden cubes, two hand puppets, a small plastic ball, one maraca, a pre-schooler’s set of scissors, six crayons of different colors, and a pen with lid. Specific indications were given to parents to administer all items at home, and they were advised not to worry if their child did not complete them all. All children were screened in their primary language, Spanish.

The review board at the Faculty of Psychology (*Facultad de Psicología*) and the General Directorate of Research (*Dirección General de Investigaciones*) of the *Universidad de la Sabana* granted ethical approval for the study (Acta CAG #1517 of 19/11/2015). Permission for data collection was granted in agreement with the legal ruling of Resolution N° 008430 of 1993 of the *Ministerio de Salud de la República de Colombia* (Health Ministry of Colombia), which sets out ethical, scientific, technical and administrative norms for research activity with human participants. At the time of screening, parents were given an information sheet describing the larger original study. Consent for participation in the research project was indicated by completion of the sociodemographic survey, prior to inclusion in the current study.

### Analysis

The analyses used average-based change statistics (ABCs), such as Cohen’s *d* or Hays’s ω2, to evaluate changes in distributions, and individual-based change statistics (IBCs), such as the Standardized Individual Difference (SID) or the Reliable Change Index (RCI), to evaluate whether each case in the sample experienced a reliable change (Clifton and Clifton, 2019; Estrada et al., 2019). The standardization of measurement differences was used to calculate the net percentage change index [i.e., 100 × (CARE score – HLL score)/(HLL score)]. Primary analyses included mixed design analysis of variance (ANOVA), with data source (i.e., direct assessment using HLL, parental report using CARE) as a within-subjects factor and screening category group (i.e., “At risk” or “Not at risk”) as a between-subjects factor, to examine consistency between HLL and CARE in determining the developmental milestones reached. Separate mixed design ANOVAs were run for each developmental domain. The decision to use a mixed design ANOVA was based on the need to compare differences between groups split on two factors: a within-subjects factor in which all participants, serving as their own matched pair, were measured in two conditions (i.e., sources of information); and a between-subjects factor in which participants were classified separately based on DS. This analytic approach follows Miller et al.’s (2017) agreement study comparing direct testing and parent reports, while also allowing evaluation of the predictive quality of CARE booklet as a screening tool.

Secondary analyses included chi-square tests of agreement on individual matched pairs of items from both primary study measures, to determine agreement at the level of specific developmental milestones. In cases where assumptions of chi-square testing were violated due to small sample sizes (i.e., less than five cases in a contingency table cell), Fisher’s exact test was used.

Using the scoring procedures described above, interviewers’ direct observations with HLL and parental reports using CARE were scored by the author and checked independently by a licensed psychologist who was a research team member. Discrepancies in scoring were resolved in face-to-face meetings of the research team and compared against hard copies of the forms, and corrections were made on the forms. Demographic form data were entered into Microsoft Excel, uploaded to a drive-in cloud storage and checked using a double-data entry procedure.

Within our main results (i.e., participant recruitment and prevalence of developmental delay), the comparative analysis for CARE using parents’ report and direct observation included:

(1) Effects of demographic variables (e.g., socioeconomic status) on overall agreement.
(2) Effects of demographic variables on the various domain scores (personal-social, language and logico-mathematical reasoning, fine motor-adaptive, gross motor skills).
(3) Overall agreement and congruence between the CARE report classification and interviewers’ direct screening classification (“At risk” or “Not at risk”), defined as the degree of correspondence between individuals’ judgments or ratings (Price et al., 2017). Inter-rater reliability (Cohen’s κ) was calculated and interpreted with the most accepted arbitrary ranges for Cohen’s κ (Landis and Koch, 1977): 0.00 – 0.20 indicates slight agreement, 0.21–0.40 fair agreements, 0.41–0.60 moderate agreement, 0.61–0.80 substantial agreement, and 0.81–1.00 indicates almost perfect agreement.
(4) Screening classification (“At risk” or “Not at risk”) differences in development domain scores between HLL and parental CARE report. Differences in counting of total “No” answers in CARE reports and “Caution” items (i.e., an age-appropriated item is not passed) in HLL were analyzed. Also, differences were reported on domain scores (personal-social; language and logico-mathematical reasoning; fine motor-adaptive; gross motor skills) for both sources of data.
(5) ROC curve area under the curve (AUC) analysis. The receiver operating characteristic (ROC) method is a commonly used paradigm in different medical and social areas to assess the performance of a diagnostic test (e.g., Schafer et al., 2014; Zanca et al., 2012). For the present study, our method requires values of two variables for each case: a truth variable (sometimes referred to as a ‘gold standard’) indicating the “At risk” status (HLL data) for each child and a decision variable indicating the CARE determination of “At risk” or “Not at risk.” The parent report in CARE is used to assign a single rating to each case (“At risk” or “Not at risk”). When the decision in CARE corresponds to the truth HLL direct observation status (“At risk”) it is called a true positive. When the decision in CARE does not correspond (i.e., “Not at risk”) to the truth HLL direct observation status (“At risk”) it is called a false negative. False positives correspond to a case when CARE reports an “At risk” condition but HLL indicates “Not at risk.” The ROC curve is a plot of true positive fraction in the sample (Sensitivity) and the complement of false positive fraction (Specificity) or 1 - Specificity. When ROC uses non-parametric estimation for diagnostic test analyses (e.g., the Wilcoxon test), it is called an “empirical ROC” (Pepe, 2003). An empirical ROC has an empirical AUC. The area under the curve has a value between 0 and 1 showing the performance of the test (CARE), with higher values indicating better test performance and 0.5 indicating randomness. For small sample sizes, the empirical AUC may change dramatically due to small perturbations and differ significantly from the expected AUC (Ma et al., 2006). An alternative to the empirical AUC is the binormal AUC (Pepe, 2003). The binormal AUC is more stable than the empirical version for small sample sizes (Ma et al., 2006). In order to present comparable empirical AUC and binormal data, I report the nominal classification analysis using previous sensitivity and specificity calculation in a web page calculation tool (VassarStats: Website for Statistical Computation) and using quantitative indices for CARE and HLL classification to plot a binormal ROC curve (Eng, 2014).
(6) Item-Level Comparison of Agreement for specific Domains. To determine agreement at the item level, a series of chi-square tests of agreement between parental reports and direct assessment was performed on individual matched item pairs. Inter-rater reliability (Cohen’s κ) and phi or Cramer’s V from the chi-square tests were reported (Bakker and Wicherts, 2011). A Cramer’s V parameter is used to compare the strength of association between any two cross-classification tables: a larger value for Cramer’s V can be considered to indicate a strong relationship between variables, with a smaller value for V indicating a weaker relationship (Price et al., 2017).
(7) Acceptability and feasibility analysis, which included six characteristics considered to influence implementation feasibility (Boggs et al., 2019): cultural adaptability, accessibility, training, administration time, geographical uptake, and clinical relevance and utility.
  When necessary, in the following analyses, assumptions of normality, homogeneity of variances, and sphericity were met, and no significant outliers were identified in our sample. Otherwise, non-normal distribution of data was analyzed with non-parametric tools (i.e., the Kruskal–Wallis test or Mann–Whitney test). An alpha level of 0.05 was adopted for all statistical tests. All statistical analyses were conducted using IBM SPSS Statistics for Macintosh, Version 25.0 (IBM Corporation, 2017).

## Results

### Prevalence of Developmental Delay

**FIGURE 4:**
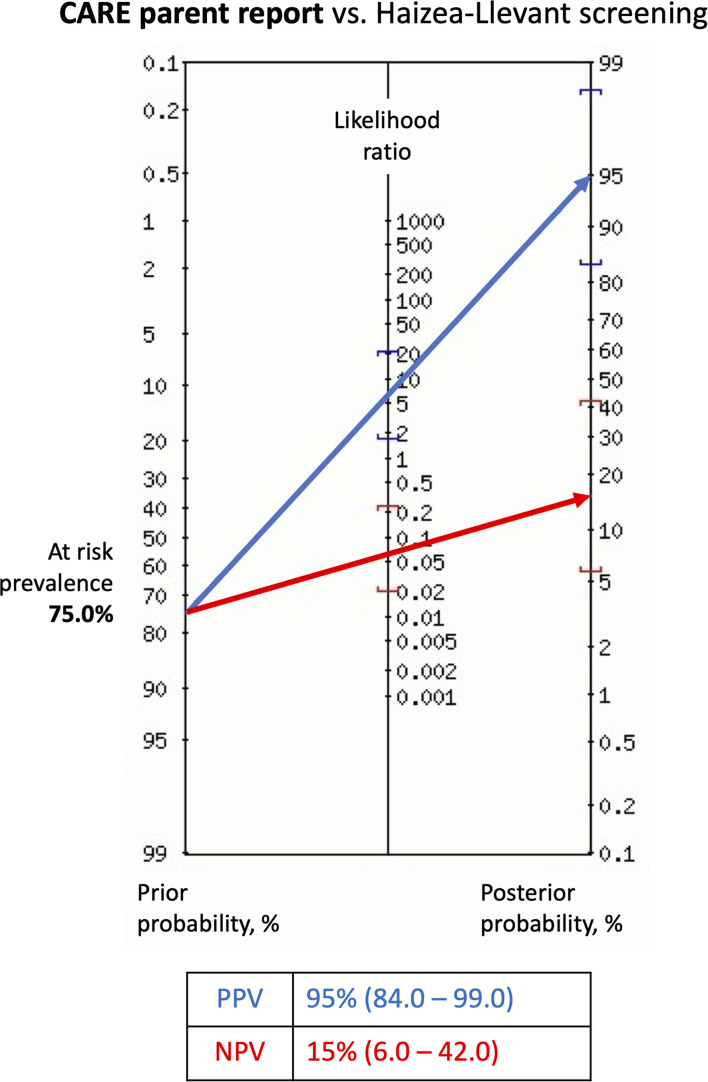
Fagan’s nomogram showing probability of children At risk after parents report using CARE booklet. Probabilities were calculated based on the screening with Haizea-Llevant table (HLL). Positive At risk diagnosis (blue arrow) refers to typical or non-specific appearance, and Not at risk diagnosis (red arrow) to atypical or negative appearance in CARE. Precision is given as 95% confidence interval. Risk prevalence is derived from the number of At risk positive and Not at risk participants after screening with HLL. LR+, positive likelihood ratio; LR–, negative likelihood ratio; NPV, negative predictive value; PPV, positive predictive value. Diagnostic test calculator (version 2010042101). Copyright (c) 2002-2006 by Alan Schwartz < alansz@uic.edu >.

Using HLL, 75% of participants were classified “At risk” (*n* = 39). The CARE booklet reported that 71% (*n* = 37) of the sample qualified as “At risk” (Figure 4). Nominal classification analysis indicated that the sensitivity proportion was high (95%, corresponding to 37 out of 39 at-risk children), as was the specificity value (85%, corresponding to 11 out of 13 not-at-risk children). Also, the positive likelihood ratio (*LR*+) was 6.17 and the negative likelihood ratio (*LR*) was 0.06.

### Effect of Demographics on Overall Agreement

Analyzing the effect of demographic characteristics in overall agreement requires individual-based change statistics (IBCs) with the net percentage change index (NET). NET is calculated by [100 × (CARE score – HLL score)/(HLL score)]. NET values indicate that the higher the difference score, the higher the probability of not agreement (Table 2). Also, negative values indicate lower score for the parental report in CARE compared to observation score using HLL (i.e., an underrated report by the parent). Differences between HLL and CARE report were higher in low SES (i.e., the second level) compared to very low SES homes. The medium-low SES was the only level at which the CARE score was lower than the HLL score.

**TABLE 2:** Raw and net percentage change index (NET) for overall scoring differences between Haizea-Llevant (HLL) and CARE.

		Haizea-Llevant overall (raw) scoring		CARE overall (raw) scoring		HLL minus CARE overall NET^+^ difference	
				
*SES*	n(%)	M	*SD*	M	*SD*	M	*SD*
Level 1 – Very low	13 (25)	0.67	*0.11*	0.68	*0.08*	3.41	*16.21*
Level 2 – Low	19 (36.5)	0.67	*0.19*	0.78	*0.11*	25.94	*40.41*
Level 3 – Medium low	14 (26.9)	0.72	*0.11*	0.70	*0.13*	–0.57	*23.31*
No data	6 (11.5)						

*^+^100 x (CARE score – HLL score)/(HLL score).*

One-way ANOVAs were then run to determine whether any sociodemographic variable had an effect on overall CARE and HLL score agreement. There was a main effect of SES on overall differences, *F*(2,43) = 6.947, *p* = 0.002, η^2^ = 0.12. *Post hoc* analyses using the Bonferroni adjusted criterion for significance and t-test when significant differences were found, indicated that differences in scores were significantly higher in low SES compared with very low SES homes, *t*(30) = –2.72, *p* = 0.011, *d* = 0.72, and with medium low SES, *t*(31) = 2.98, *p* = 0.006, *d* = 0.81.

No significant effect of other sociodemographic variables, including whether the child was a boy or a girl, was found on overall scoring differences between data sources (HLL vs. CARE) in the total sample.

### Effect of Demographics on Domain Scores

Individual difference scores were calculated for analyzing the effects of demographic characteristics in every developmental domain assessed with HLL and CARE screening. The net percentage change index (NET) was calculated by subtracting each age-equivalent standardized individual CARE score from the age-equivalent standardized individual score in the corresponding developmental domain (Table 3).

**TABLE 3:** Median and data spread (Interquartile range-IQR) for the Net percentage change index (NET) between scores for Haizea-Llevant (HLL) and CARE report by developmental dimensions.

	*Personal-social domain*		*Language and logico-mathematical reasoning*		*Fine motor-adaptive domain*		*Gross motor domain*	
	Median	*IQR*	Median	*IQR*	Median	*IQR*	Median	*IQR*
***Sex***								
Male	–12.7	*18.9*	–11.2	*16.9*	–15.2	*21.4*	–10.0	*27.6*
Female	–12.8	*12.5*	–8.5	*10.8*	–11.4	*13.4*	–13.3	*11.3*
***Working mother status***								
Employed	–13.5	*19.6*	–8.6	*16.6*	–13.2	*19.4*	–14.6	*23.2*
Unemployed	–9.4	*13.1*	–16.6	*18.4*	–11.7	*28.0*	–19.9	*38.9*
***SES***								
Level 1 – Very low	–15.4	*27.0*	–7.7	*15.4*	–15.2	*9.7*	–16.1	*9.4*
Level 2 – Low	–8.2	*13.1*	–16.8	*15.6*	–8.1	*32.6*	–9.3	*40.7*
Level 3 – Medium low	–16.5	*28.8*	–8.6	*9.0*	–8.3	*21.6*	–6.9	*35.5*

Raw differences or standardized Individual Differences (SID) with negative values indicate lower score for the parental report in CARE compared to observation score using HLL (i.e., underrated report by parent). All medians with negative values indicate a central tendency with lower scoring in CARE report compared with HLL’s scoring. Differences were higher in Personal-social and Gross motor domains for girls. Language and logico-mathematical reasoning and Fine motor-adaptive domains scorings has higher differences for boys. Working mothers had higher differences in Personal-social and Fine motor-adaptive for Employed status. Language and logico-mathematical reasoning and Gross motor domains scorings has higher differences for Unemployed status. Also, differences were higher in Personal-social domain for Medium low SES and in Language and logico-mathematical reasoning for Low SES (i.e., the second level). Fine motor-adaptive and Gross motor domains scorings have higher differences for Very low SES compared with other SES levels.

A Mann–Whitney test indicated a significant effect of working-mother status, with higher difference for employed (*Median* = –13.2) than unemployed mothers (*Median* = –11.7) on HLL and CARE scorings in the fine motor-adaptive domain, *U* = 114.5, *p* = 0.02, *r* = 0.33.

No significant effect of any other sociodemographic variables was found on developmental domains differences between data sources (CARE vs. HLL), suggesting that parents did not significantly differ in their ratings of child skills using CARE compared to direct testing with HLL in the total sample.

### Overall Agreement Between Haizea-Llevant and CARE Screening Classification (“At Risk,” “Not at Risk”)

When comparing the classification outcomes of CARE booklet with the HLL, the overall agreement was 92% (by accuracy). Cohen’s κ was calculated to determine if there was an agreement between the nominal screening classifications (“At risk” or “Not at risk”) in HLL and CARE. There was almost perfect agreement between the two classifications data, κ = 0.810 (95% *CI* –0.973, –0.988), *p* < 0.0001.

### Screening Classification (“At Risk,” “Not at Risk”) Differences in Delay and Caution Items Between Haizea-Llevant and CARE

Table 4 presents descriptive statistics of overall performance on items (i.e., Delays and Cautions) and nominal classification (i.e., “At risk” or “Not at risk”) using HLL and parents’ reports using CARE. In the HLL reports, more items were reported as Cautions than Delays. The same was true for CARE reports in “Not at risk” participants. Contrary, Delays were four times more likely to be reported in “At risk” children when using the CARE report.

**TABLE 4:** Delays and Cautions for nominal classification groups using Haizea-Llevant (HLL) and CARE.

	*n* (%)	Items in Delay		Items in Caution	
		Median	*IQR*	Median	*IQR*
***HLL-Observation***					
At risk	39 (0.75)	1.0	*2.0*	3.0	*3.0*
Not at risk	13 (0.25)	0.0	*0.0*	1.0	*0.0*
***Using CARE report***					
At risk	39 (0.75)	4.0	*3.5*	1.0	*4.5*
Not at risk	13 (0.25)	0.0	*1.0*	1.0	*1.0*

A Mann–Whitney tests indicated a significant difference in HLL observations, such that the “At risk” group presented a greater number of Caution items (*Median* = 3) than the “Not at risk” group (*Median* = 1), *U* = 66.0, *p* < 0.001, *r* = 0.56. Similarly, “At risk” children presented a greater number of Delay items (*Median* = 4) than the “Not at risk” group (*Median* = 0), *U* = 85.5, *p* < 0.001, *r* = 0.50.

### Screening Classification (“At Risk,” “Not at Risk”) in Development Domain Scores for Haizea-Llevant and CARE

Standardized individual scores were calculated for analyzing developmental dimensions (i.e., Personal-social domain) and nominal classification (i.e., “At risk” or “Not at risk”) using both HLL and CARE (Table 5). Differences were greater in HLL classification in the personal-social and language and logico-mathematical reasoning domains for “Not at risk” children. Also, same children (HLL classification: “Not at risk” children) had a higher CARE report scoring than their HLL score in the gross motor domain. Fine motor-adaptive scorings had higher differences for “At risk” children classified using HLL observation. Greater differences with higher CARE report scoring than HLL score were seen for “Not at risk” children in all domains.

**TABLE 5:** Median and data spread (Interquartile range-IQR) for the Net percentage change index (NET) between scores for Haizea-Llevant (HLL) and CARE report by developmental dimensions.

	*Personal-social domain*		*Language and logico-mathematical reasoning*		*Fine motor-adaptive domain*		*Gross motor domain*	
	Median	*IQR*	Median	*IQR*	Median	*IQR*	Median	*IQR*
***HLL-Observation***								
At risk	*0.20*	*0.98*	*0.00*	*1.74*	*–0.15*	*1.51*	*0.00*	*1.46*
Not at risk	*–0.29*	*2.83*	*–0.59*	*1.25*	*0.13*	*1.85*	*0.73*	*0.00*
***Using CARE report***								
At risk	*–0.30*	*1.12*	*–0.22*	*1.06*	*–0.02*	*0.00*	*0.05*	*0.00*
Not at risk	*0.89*	*0.00*	*1.03*	*0.43*	*0.81*	*0.00*	*0.73*	*0.00*

A Mann–Whitney test indicated that scores on the CARE report in the personal-social domain were lower for the “At risk” group (*Median* = 0.7) than for the “Not at risk” group (*Median* = 1.0), *U* = 82.5, *p* = 0.001, *r* = 0.52. No significant difference was found between “At risk” or “Not at risk” groups on personal-social domain scores for direct testing with HLL. Comparing scores in language and logico-mathematical reasoning, using a Mann–Whitney test, indicated that on CARE report scores were lower for the “At risk” group (*Median* = 0.7) than for the “Not at risk” group (*Median* = 1.0), *U* = 74.0, *p* = 0.001, *r* = 0.53. No significant difference was found between “At risk” or “Not at risk” groups on language and logico-mathematical domain scores for direct testing with HLL. Also, a Mann–Whitney test indicated that score in fine motor-adaptive domain on CARE report was lower for the “At risk” group (*Median* = 0.8) than for the “Not at risk” group (*Median* = 1.0), *U* = 118.5, *p* = 0.01, *r* = 0.42. No significant difference was found between “At risk” or “Not at risk” groups on fine motor-adaptive domain scores for direct testing with HLL in the total sample (data not shown). Score in gross motor domain on CARE report, a Mann–Whitney test, indicated that was lower for the “At risk” group (*Median* = 0.80) than for the “Not at risk” group (*Median* = 1.0), *U* = 110.5, *p* = 0.01, *r* = 0.45. Likewise, scores in gross motor domain on direct testing with HLL was lower for the “At risk” group (*Median* = 0.75) than for the “Not at risk” group (*Median* = 1.0), *U* = 72.5, *p* = 0.05, *r* = 0.30.

### Receiver Operating Characteristic Curve: Area Under the Curve

**FIGURE 5:**
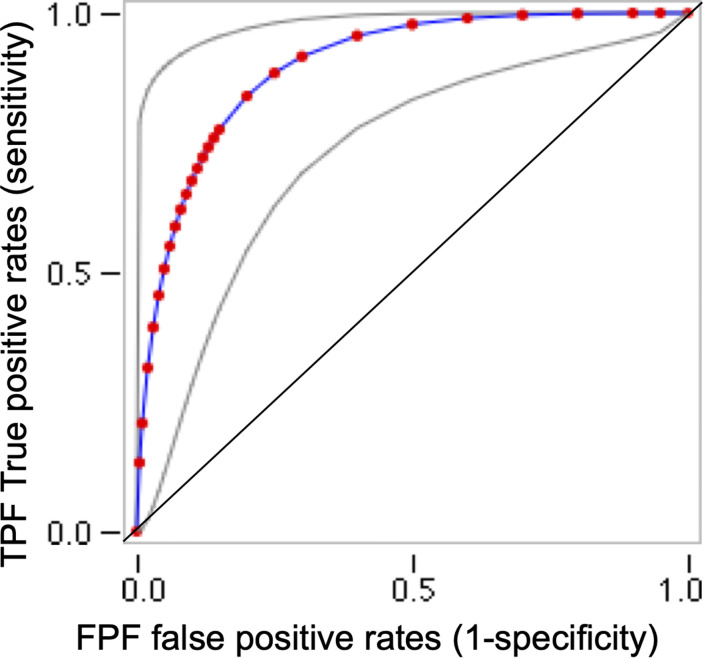
Receiver operating characteristic (ROC) binormal curve for CARE and Haizea-Llevant classification for the total sample (*n* = 52). This ROC curves plot use web-based calculator for ROC curves (http://www.jrocfit.org). Gray lines indicate 95% confidence interval of the fitted ROC curve. ROC analysis plot for each possible cut-off points of the relevant CARE scale, the true-positive proportion (sensitivity = 95%) against the false-positive proportion (1– specificity). A perfect test would have an area under the curve (AUC) of 1 and the curve would pass through the upper left corner of the plot (100% sensitivity, 100% specificity). In this study, Trapezoidal (Wilcoxon) area/AUC = 0.89 (*SE* = 0.04) and the Area under the fitted curve (*Az*) = 0.90 (*SE* = 0.052).

When performing an empirical ROC-curve analyses in the total sample (*n* = 52), the area under the curve (AUC) is 0.894 (Trapezoidal Wilcoxon area) with a higher Youden index of 0.860 (Supplementary Table 1). Otherwise, a binormal ROC curve (Figure 5) uses quantitative index for CARE and HLL classification as a truth variable indicating the “At risk” status for each child. The Area under the fitted curve (*Az*) in the binormal curve is 0.899.

Youden J indexes (Supplementary Table 1) are reported because they indicate the maximum potential effectiveness of CARE scoring, and act as a common summary measure of the ROC curve (Ruopp et al., 2008).

### Item-Level Comparison of Agreement for Specific Domains

Given the small group sizes when the sample was split by demographic variables, item level analyses were conducted on the full sample instead of separately for each screening group. Table 6 shows the mean proportions of correct items in the HLL and CARE reports. An important aspect to note is the asymmetry in the number of participants due to the application of HLL to specific ages and the delivery of CARE to the general sample. After descriptive data, the agreement at the item level was determined with a series of chi-square tests, performed on individual matched item pairs across HLL and CARE scores and developmental dimensions.

**TABLE 6:** Media and standard deviation (SD) for assertive observation or reports in Haizea-Llevant (HLL) and CARE by items in developmental dimensions.

	HLLevant			CARE		
*Personal-social domain*	*n*	*M*	*SD*	*n*	*M*	*SD*
Help in House	4	1.00	*0.00*	9	0.67	*0.73*
Feed doll	7	0.86	*0.76*	9	0.89	*0.67*
Remove Garment	12	1.00	*0.00*	9	0.89	*0.67*
When he or she play with dolls, he/she performed a play like a script or short tale with their dolls or toys?	17	0.94	*0.49*	52	0.92	*0.36*
Put on clothing	30	0.56	*1.00*	52	0.77	*0.74*
Did he/she suggest or show when need to go to the toilet?	17	1.00	*0.00*	50	0.88	*0.27*
Did he/she answer if he or she is a boy or a girl?	30	0.78	*0.86*	52	0.90	*0.50*
Dress, no help	26	0.41	*1.02*	52	0.71	*0.85*
Did he/she play with an adult using hand puppets?	31	1.00	*0.68*	52	0.87	*0.41*
Prepare cereal (In Spanish this item is open to more food than cereals)	24	0.64	*0.95*	43	0.84	*0.67*
Draw a person	16	0.44	*0.91*	43	0.53	*0.95*
***Language and logico-mathematical reasoning***						
Name __ Pictures (6 pictures)	5	0.87	*1.10*	9	0.67	*0.88*
Know 2 actions	5	0.63	*1.10*	9	0.78	*0.71*
Combine words	5	0.40	*1.10*	9	0.56	*0.87*
Name __ Pictures (5 pictures)	9	0.56	*1.05*	9	0.89	*0.33*
Use of 3 Objects	10	0.40	*0.97*	9	0.89	*0.33*
Speech half understandable	12	0.40	*0.90*	26	0.89	*0.33*
Did he/she point the dog correctly? (memorize an image)	19	0.70	*0.96*	35	0.85	*0.59*
When he or she speaks use pronouns?	29	0.28	*0.94*	9	0.97	*0.17*
Did he/she count aloud two consecutive numbers?	27	0.43	*1.02*	52	0.79	*0.71*
Name __ Pictures (10 pictures)	33	0.68	*1.01*	52	0.96	*0.19*
Did he/she use “to be” in a phrase?	33	0.30	*1.00*	52	0.90	*0.50*
Pick longer line	38	0.42	*1.01*	52	0.90	*0.55*
Speech all understandable	37	0.51	*0.99*	51	0.85	*0.62*
Identify colors	36	0.50	*0.96*	43	0.79	*0.82*
Did he/she realize no-connected actions?	39	0.63	*0.97*	43	0.88	*0.55*
Name colors	27	0.54	*0.90*	43	0.79	*0.68*
Opposites – morning/afternoon	23	0.36	*0.93*	43	0.79	*0.68*
Did he/she tell stories?	16	0.62	*0.25*	43	0.63	*0.92*
Did he/she repeat a complete phrase?	12	0.41	*0.51*	43	0.67	*0.83*
Did he/she recognize numbers (Arabic writing numerals)?	12	0.42	*0.52*	43	0.56	*0.92*
***Fine motor-adaptive domain***						
Put Block in Cup	6	0.94	*0.00*	9	1.00	*0.00*
Tower of 4 cubes	9	0.62	*0.00*	9	1.00	*0.00*
Thumb-finger grasp (grab a pencil)	16	0.54	*1.03*	52	0.88	*0.46*
Copy a circle	30	0.00	*1.02*	52	0.87	*0.61*
Did he/she imitate a bridge with 3 cubes?	37	0.00	*1.01*	52	0.87	*0.57*
Did he/she fold a paper sheet?	30	0.74	*0.82*	44	0.73	*0.69*
Did he/she use scissors to cut a paper sheet?	26	0.59	*0.98*	44	0.77	*0.64*
Copy a square	19	0.53	*1.01*	44	0.64	*0.82*
Did he/she imitate a door with 5 cubes?	19	0.79	*0.84*	44	0.73	*0.69*
***Gross motor domain***						
Walk down steps	4	0.58	*0.00*	9	1.00	*0.00*
Kick ball forward	4	0.58	*1.00*	9	1.00	*0.00*
Broad jump	17	0.79	*0.87*	52	0.85	*0.58*
Balance Each Foot 5 s	28	0.00	*0.92*	52	0.75	*0.75*
Jump up	29	0.25	*0.82*	52	0.79	*0.64*
Did he/she jump backwards?	22	0.76	*0.46*	52	0.69	*0.66*
Balance each foot 1 s	18	0.79	*0.57*	44	0.75	*0.69*

Several chi-square tests indicated, overall, somewhat mixed item-level agreement findings for every domain. The proportion of items with significant agreements was higher in personal-social (7 out of 11: 63%) and language and logico-mathematical reasoning (14 out of 20: 70%) than the proportions in fine motor-adaptive (5 out of 9: 55.5%) and gross motor skills (3 out of 7: 42.8%). However, nearly all scores for items accrued in one quadrant of the chi-square contingency table. Under that condition there are key limitations to adequate interpretation for Kappa values for agreement between data sources. That is a reason to report Cramer’s *V* (Gingrich, 2004), which is used to compare the strength of association between any two cross-classification tables. Tables which have a larger value for Cramer’s *V* can be considered to have a strong relationship between the variables, with a smaller value for V indicating a weaker relationship (Gingrich, 2004).

#### Personal-Social Domain

For items assessing personal-social domain (e.g., “Help in house”), there was more significant agreement than non-agreement between parental report and direct testing (Supplementary Table 2). However, on some items measuring-agreement continuity is expected, because some activities will use the same objects in a trajectory of increasing complexity in interactions with adults or peers. Items like “Feed doll” and “When he or she plays with dolls, he/she performed a play like a script or short tale with their dolls or toys?” or “Did he/she play with an adult using hand puppets?” are examples of the expected trajectory. The expected trajectory apparently requires more complex developmental skills that affect the agreement level. Another example is “Remove garment” and “Put on clothing” or “Dresses, without help.” For those items, parents mostly reported that the child had the skill, but it was not seen on direct testing. Finally, a significant disagreement (κ ≤ 0) between CARE and HLL direct testing was found in “Did he/she suggest or indicate needing to go to the toilet?”, showing that this particular behavior was more often seen in direct assessment than reported by parents.

#### Language and Logico-Mathematical Reasoning

For items assessing language and logico-mathematical reasoning skills (e.g., “Combine words”), there were more items in significant agreement than items with non-agreement between parent report and direct testing (Supplementary Table 3). However, as in the personal-social domain, there were items where measuring-agreement continuity was not obtained, e.g., “Did he/she count aloud two consecutive numbers?” and “Did he/she recognize numbers (Arabic numerals)?”. Also, perceptual and contextual discrimination skills were not in agreement (i.e., parents reported that the child could “Pick longer line” and recognize “Opposites - morning/afternoon” more often than seen on direct assessment). Likewise, some expressive language items had no significant agreement (i.e., “Did he/she use ‘to be’ in a phrase?”; “Did he/she repeat a complete phrase?”).

#### Fine Motor-Adaptive Domain

For items assessing fine motor-adaptive skills (e.g., make a “Tower of four cubes”), there was almost the same number of items in significant agreement than those without significant agreement between parent report and direct testing (Supplementary Table 4). However, as with previous domains, there were items where measuring-agreement continuity was not obtained (i.e., “Tower of four cubes” vs. “Did he/she imitate a bridge with three cubes?”, and “Copy a circle” vs. “Copy a square”).

#### Gross Motor Domain

For items assessing gross motor domain (e.g., making a “Wide jump”), there were more items with no significant agreement than items with significant agreement between parent report and direct testing (Supplementary Table 5). As in previous domains, there were items where measuring-agreement continuity was not obtained (i.e., “Wide jump” and “Jump up”).

### Acceptability and Feasibility

The rating criteria in Boggs et al. (2019) for mentioned characteristics in screening tools were applied to the CARE reports. Validity and reliability analysis was presented in previous sections. According to Boggs et al. (2019), CARE presented several characteristics in rating levels between 0 and 3, indicating a good consideration for scalable studies (Table 7).

**TABLE 7:** CARE characteristics according to early child development measurement tool accuracy and feasibility for use in routine programs criteria by Boggs et al. (2019).

	Boggs level description	Observation about CARE
Cultural adaptability, Rating: 3	Easy modification of items, materials and procedures.	All items have a particular space for annotations a personalize descripted instructions or activities. The modification of items, materials and procedures will be fitted according inhouse context. Pictures and words are widely understood for specific participants with low academic level.
Accessibility, Rating: 2	Tool, administration, scoring and interpretation, adaptation and training resources all available open access online with no intellectual property restrictions, minimal cost to tool and/or equipment (≤US$10 per child), no app available.	CARE is online available at https://monitoreoencasa.weebly.com/The toys and materials delivered with the printed booklet cost less than 7 GBP per child.
Training, Rating: 3	Brief (≤1 h), minimal (i.e., non-specialist worker can train non-specialist worker), no certification requirement.	Parents only received a less than 3 min video instruction (https://youtu.be/Y5864iGCvG8); research team are undergraduate students and do not receive specialized instruction for cooperation or answer questions coming from parents.
Administration time, Rating: 2	>15 to ≤30 min, minimum to moderate scoring.	CARE is planned to apply at home. A direct question about accumulated time when the booklet is returned to research team indicates less than an hour throughout a 1 month.
Geographical uptake, Rating: 0	Used in one country only.	Only used in Colombia.
Clinical relevance and utility, Rating: 3	Easy interpretation, clear threshold for action and structure for counseling response and contextually appropriate referral.	CARE is intended to use it as referral for clinical surveillance and motive observations an interaction between caregivers and children at home. All individuals had a one-page results, as a guide for educative action and understandable by caregivers and CC workers in the individual report returned as feedback to participants.

## Discussion

The CARE booklet featured in this study aims to monitor and support parents’ interactions for enhancing children’s development and identify developmental difficulties. The previous phases of this study include the conceptualization and consolidation of CARE components related to the Haizea-Llevant DS table (HLL). The monitoring component of CARE is central to the current study reported here, in particular an examination of its sensitivity and specificity in a small sample of vulnerable families in Colombia. The sample of families and children recruited from a community children’s center in Colombia’s capital, Bogotá, was similar to those for which similar screening tools are designed and standardized in LMIC populations (Faruk et al., 2020).

Firstly, a positive characteristic of CARE is in the level of engagement shown for a measurement tool relating to a cognitive intervention. Following a meta-analysis for commitment of parental involvement (Haine-Schlagel and Escobar-Walsh, 2015), completion of tasks in cognitive interventions had a range of 19–89% in participants. The effective users of the CARE booklet in this study were the 85.2% of receivers who used it for 1 month at home. The high level of CARE report use has considerable positive implications for the whole monitoring, screening and surveillance cycle to track a child’s developmental progress (Faruk et al., 2020), known as the detection-intervention-prevention continuum.

Second, concerning the prevalence of developmental delay, our procedure to recruit participants after a first screening may have affected the high level of delay found (75%), raising concerns for more wide-ranging recruitment in an experimental field procedure using CARE as a screening tool. However, recent studies reported low delay prevalence in DS (Ozturk-Ertem et al., 2019) and the higher prevalence in our study must be interpreted with caution. If excluding participants to receive the CARE booklet after first screening is a recruitment bias, it is an opportunity for methodological improvement since several barriers to the identification of developmental delay using tools adapted for LMIC have recently been reported (Faruk et al., 2020). Indeed, other screening studies include samples that did not share comparable sociodemographic characteristics to our participants, such as lower socioeconomic status (Murphy et al., 2020). According to the expressed aims of the current study, next discussions comprehend the specific results.

### Consistency Between CARE and Haizea-Llevant Classification and Scores in Developmental Domains

Overall, the results suggest that parental observation of different child abilities reported in the CARE booklet did not differ significantly from direct assessment using HLL, and results were generally stable across screening classification groups (i.e., overall agreement by accuracy: 92%). Also, the effects of demographic variables on agreement between parent report and direct assessment of child are fundamental for decisions on future research and interventions after the COVID-19 pandemic. Differences for lower socioeconomic status and working-mother status indicated a need for better tracking of interactions related to parenting employment and individual developmental trajectories when those demographic conditions are present in LMIC populations (Campaña et al., 2020). Language and mathematical reasoning and fine motor skills were the two skill areas most affected by SES conditions in our data, in common with previous studies of early childhood (Justice et al., 2019). Some barriers connected with caregivers serving as informants of their own interactions’ quality relate to parental distress around parent–child interactions. CARE DS might diminish parental stress or other contingent conditions associated with dysregulated parent–child interactions and reported in vulnerable or impoverished conditions (Justice et al., 2019). However, SES is not defined solely by economic poverty, and more research is need in order to clarify the issue of scarcity in child–parent interactions (Guan et al., 2020).

Altogether, these findings suggest that both CARE reports and direct testing are appropriate forms of child DS. However, this study has an advantage over other comparisons with agreement analyses, including Miller et al. (2017): 100% of items in the parental reports (the CARE booklet) were comparable with the items included in the direct screening measurement. Indeed, Miller et al. (2017) only compared 12 out of 381 items (3.15%) for the Vineland Adaptive Behavior Scales (Survey Interview Form; Sparrow et al., 2005) and 12 out of 91 items (13.2%) for the Mullen Scales of Early Learning (Mullen, 1995). The good agreement shown in our results suggests that parents are generally reliable reporters of child abilities. When comparing agreement between “At risk” classification and scores on CARE and HLL (see Tables 4, 5), across the domains of personal-social skills, language and logico-mathematical reasoning, fine motor-adaptive and gross motor skills, CARE demonstrated discriminatory potential that was as good as that provided by the HLL direct observations.

In particular, while HLL is a better detector for Cautions, CARE demonstrated better discrimination for Delays. Furthermore, all developmental domains had differences in nominal classifications in the “At risk” and “Not at risk” groups using CARE, but only in the gross motor skills dimension using HLL. A next step in the optimal design process for CARE should be a comparison with other tools in order to establish wide discriminatory characteristics in a Field Testing-Analysis-Revision framework (Nadeem et al., 2016).

### Item Level Consistency Between CARE and Haizea-Llevant

Overall, the proportion of items in agreement were higher for personal-social and for language and logico-mathematical reasoning compared to the proportions for fine motor-adaptive and gross motor skills. The obvious answer to explain this discrepancy would be the time dedicated to observation of interactions. CARE gives parents 1 month to screen their children constantly on four developmental dimensions. Unfortunately, an explicit limitation is in the lack of analysis for any difference regarding the time it takes for parents to complete the CARE booklet. That means a limitation in determining the effect of the whole time dedicated to use and return CARE, as it could be done in a day, during a week or over the whole month. However, these long-lasting observations with the screening activities in CARE relating to fine motor-adaptive and gross motor skills might increase the disagreement with the short-term observations using HLL, given the accumulation of time and opportunities for reporting motor interactions at home. Otherwise, a significant disagreement (κ ≤ 0) between CARE and HLL direct testing was found in “Did he/she suggest or indicate needing to go to the toilet?”, with this particular behavior more often seen in direct assessment than reported by parents. The autonomy levels expected in the test environment are different in the Children’s Center compared to the child’s home. Also, such items will be subject to parents’ interpretation according to the cultural context (Schiariti et al., 2021). In this specific case, the lack of autonomy assigned to going to the toilet, and other social items, could result from parents assuming that a child cannot perform age-appropriate tasks without having actually observed these in detail at home (Miller et al., 2017). CARE screening might demand attention to behaviors, skills and performances that routinely are included in at-home interactions and excluded in the report. The attentional demands of routine interactions between parents and children were recently included in an analysis of associations between high levels of cognitive stimulation in the home and increased screening scores for children in low-SES conditions (Slemming et al., 2021). Specifically, they analyzed this under the so-called “standard model” of consecutive *knowledge* → *stimulation* → *development* (Bornstein, 2015; Britto et al., 2017; Cuartas et al., 2020).

The *knowledge* → *stimulation* → *development* (*K*→*S*→*D*) model acts like a “cascade” of processes and outcomes, involving parenting attributions and supportive parenting, and concluding in the child’s externalizing behavior. In the *K*→*S*→*D* model, the testing of any particular child’s skills by observation has specific challenges for parents and even for professional experts in child development, despite their favorable knowledge and attitudes (Jain et al., 2021) and appropriate healthcare organizational setup (Sheeran et al., 2020). Child non-compliance reduced attention and interest in calls for interaction, and the unfamiliar framework for direct reports at home might affect the success of testing. Recent research confirms the relevance of responsive parental behavior and child’s interactive engagement for positive developmental trajectories in children with significant cognitive and motor developmental delay (Van Keer et al., 2020). The level of attention from parents, and the initiation of interactions by children, might explain why the frequency, continuity and quality of interactions at home affect positive parental reports when interaction is not complex, but disagrees with external observation when complexity in interactions is higher and is not capable of full reporting through the screening measurements. In our data, the disagreement levels were specifically noted in fine and gross motor skills (i.e., proportion of items without significant agreements: 57.2%), as we expected and was suggested before by Miller et al. (2017).

Moreover, the *K*→*S*→*D* model implies that parents might recall whether a skill milestone had effectively been reached, before confirming this through observation. If the CARE delivery is not enough for changing parental knowledge of stimulating interactions and consequently affecting children’s outcomes, a pre-post study might indicate the need for a new design, beyond CARE delivery as an intervention with screening tools.

### Diagnostic Characteristics and Performance of CARE as a Tool for Developmental Screening

Receiver operating characteristic analysis results indicated that CARE is a satisfactory tool for screening diagnostics and might help to build a quantitative index for better and faster classification of an “At risk” status in children aged 24–59 months. Our data offers complete diagnostic performance for a screening tool, surpassing the limitations of other tools designed and developed in LMIC (Faruk et al., 2020), such as the Child Language Test in Phonology, Vocabulary, Fluency and Pragmatics (ABFW), the Developmental Assessment Scales for Indian Infants (DASII), and the Rapid Neurodevelopmental Assessment (RNDA; Juneja et al., 2012; Khan et al., 2013; Dias et al., 2020). There is no ROC analysis of ABFW, DASII or RNDA to compare with our data. However, the sensitivity and specificity (95 and 85% respectively) of CARE were higher than for another tool validated against the Denver Developmental Screening Test, namely, the Trivandrum Developmental Screening Chart (TDSC). The TDSC had an overall sensitivity and specificity of 66.7 and 78.8%, respectively. The diagnostic characteristics of CARE are highly trustworthy compared to other screening tools designed for long observation periods by parents. However, due to the limitations set out in the next section, we cannot say that CARE might be better than the Guide for Monitoring Child Development (GMCD) or other tools targeted at early ages or specific developmental domains, such as social-emotional or self-help subscales (Faruk et al., 2020).

### Pilot Validity of CARE for Research and Intervention With Institutional Community Participants

The CARE booklet, and other screening tools administered by parents, might act like home-based records (HBRs). Such records do not replace clinical or scientific intervention, but can run in parallel with other existing or subsequent screening tools for optimal health and educational system interventions (Mahadevan and Broaddus-Shea, 2020). The CARE booklet shows similar conditions for delivery as HBRs, with rigorous reliability and agreement results. Also, CARE content and design had enough cultural adaptability to follow the Nurturing Care Framework and could be administrated in programs like FAMI for rural families in Colombia (Milner et al., 2019). Following the standards of Boggs et al. (2019) for screening tools, the accessibility of CARE might be diminished by the fact that there is no digital app for it available. However, this might not be true for families with lower resources or in some geographical regions, who may not access the internet. A first step considering the relevance of Boggs et al. (2019) but forgetting the focus on vulnerable and limited resources for families in poverty is in an online information-delivery through a beta webpage with a digital version of CARE^1^. The availability of CARE in electronic format limits the delivery for the focused families in the present study. However, it will contribute to even easier access and optimal conditions for training and administration time in families and health systems having non-limited connection or access to the internet.

Finally, as a preliminary conclusion, CARE may be an efficient, cost-effective screening instrument for children between aged 24–59 months who are at risk of not reaching all their cognitive potential because of social and economic limitations. The clinical relevance and utility of the accurate and efficient classification obtained with tools like CARE might be successfully included in health systems and surveillance routines for DS in the detection of delay, and can be useful for identification and electronic records as well (Vitrikas et al., 2017; Gellasch, 2019). Developmental monitoring and screening processes in LMIC should use tools like CARE for detecting and increasing early intervention referrals, assessments and eligibility for the children who need it most (Barger et al., 2018; Goldfeld and Yousafzai, 2018). CARE not only shows the desired sensitivity-specificity values, but also provides information on cultural adaptation with respect to the communities that use Children’s Centers for vulnerable families in Colombia. The reported diagnostic and screening characteristics also most likely resulted in the high level of acceptance of the screening process (75.1%), which is crucial for the success of a large-scale surveillance program. However, attention to the limitations of this study and the possibility for further research is needed to evaluate its potential for population screening and monitoring, and its cost-effectiveness as a public health measure.

### Limitations in CARE Screening and Diagnostics Characteristics

The lack of data about the clinical status of parents using CARE helps to maintain the consideration of parental discrepancy in reports as an essential source of information, given the assuming norm that parents are uniquely positioned to observe and interact with children in various situations at home (Bennetts et al., 2016; Miller et al., 2017; Jeong et al., 2019). However, the results of the item analysis require an explanation of certain disagreements and inconsistencies. The data appear overall to have no systematic pattern of disagreement in the consideration of items by domains (i.e., proportion of items with significant agreements, personal-social: 63%, language and logico-mathematical reasoning: 70%, fine motor-adaptive: 55.5%, gross motor skills: 42.8%), but some disagreements (e.g., “Copy a circle”: κ = 0.015, *p* = 0.72; “Copy a square” κ = 0.125, *p* < 0.01) show a truncated continuity in the screening process by parents when the nature of the activities increases the complexity in some domains. The *K*→*S*→*D* model explain the probability of memory and recall use for parent’s report, but do not resolve this issue in future and scalable applications of CARE. As indicated before, this a pilot phase of CARE for optimizing the design following the components of Nadeem et al. (2016) and several other limitations in the present study might be addressed before subsequent field testing.

Also, our standardized DS tool, the HLL has its own limitations. First, the last reported use and correction was normed a decade ago (Rivas et al., 2010) and it is thus less up-to-date than other early DS tools (Boggs et al., 2019). Second, like any other screening test, CARE only allows for a ‘snapshot’ of a child at one time point, limiting the ability to capture the full range of a child’s functioning. The CARE snapshot might lead to interpreting a false classification or disagreement at item level (compared to the HLL observation) as “parental error” (Miller et al., 2017, p. 12). Miller et al. (2017) argued that it cannot be systematically ascertained whether a child’s behavior during the evaluation was typical of his or her home behavior. An alternative to the “error” explanation is a hypothesis related to the effects of the psychology of scarcity (Shah et al., 2012, 2015, 2018; Camerer et al., 2018). This argument might be called the “scarcity of parental interactions” argument as opposed to the error argument (Miller et al., 2017). For the other kind of disagreements, “when a parent reports that a child has a skill, yet the skill is not seen on direct assessment” (Miller et al., 2017; p. 12), parents might use two strategies to report using CARE: (a) recall or memory of interaction events, and (b) direct subsequent observations of their interactions with children. A limitation on analyzing these disagreements is in the lack of more invasive research and evaluation techniques in this study, with a clear suggestion of including home-visit observations or home-recorded videos.

### Limitations in the Study Design and Further Studies

Using CARE as a screening tool have the potential to activate alerts for early cognitive delay that reassure clinicians and families of further specialized and controlled developmental evaluations, and that act as a screen for the presence of such delay across four developmental dimensions. The high predictive ability of CARE (Sensitivity = 95%, Specificity = 85%) in typical children of our sample but at risk of not reaching all their cognitive potential because of social and economic limitations allow considerations for future studies to investigate the measurement of the social skills for the detection of possible early signs of autism spectrum disorder (ASD) in toddlers.

However, further research is necessary to evaluate if limitations related to the sample size and sampling methodology might invalidate these possibilities, such as adding an analysis report on whether the sensitivity and specificity values obtained in CARE vary with children’s age. Consequently, the overall results and item analysis of the current study should be interpreted with caution. All suggested diagnostic properties and patterns of agreement and disagreement in the data should be considered exploratory.

Most notably, the final sample and the small within-group numbers demonstrate the effects of demographic variables and item-level results that might be corrected with a large and randomized selected sample. Future research is needed to examine specific skills that are under- or over-reported, and the influence of parents and interviewers’ characteristics, like the information on the clinical status of the parents, on the agreement between parent reports and direct testing.

Finally, screening and diagnostics using parent reports as part of long-reach monitoring for social and cognitive developmental status require an examination of engagement and attrition levels of the participants. Previous literature reported parental engagement by an average completion rate across all cognitive intervention sessions (Haine-Schlagel and Escobar-Walsh, 2015). The average rate is for 49% of participants to abandon the process before cognitive interventions, with a range from 19 to 89%. Haine-Schlagel and Escobar-Walsh’s (2015) research indicates that in our case, the 14.9% not returning CARE forms (i.e., attrition) for a non-clinical intervention is very good, but would still reward future inquiry about this issue. Recent studies dedicated to Spanish-monolingual US Latino parents’ engagement in an evidence-based program focused on promoting sensitive, responsive parenting for socioeconomically disadvantaged families (So et al., 2020) indicated distinct barriers (e.g., employment challenges, health-related challenges) and facilitators (e.g., knowing other mothers in the group, interest in the program topics), none of which were explored in the current study with CARE.

Further studies should examine whether direct observation at home affects individual development status, and what differences might appear when CARE is not only delivered as a screening tool but structured as an intervention. A comparison with structured interventions will provide a preliminary idea of whether instruments like CARE affect children’s outcomes simply by giving caregivers indications to observe and report a broad spectrum of developmental interactions, as do the Guide for Monitoring Child Development (GMCD) and other tools used in global programs (Faruk et al., 2020).

## Data Availability Statement

The raw data supporting the conclusions of this article will be made available by the authors, without undue reservation.

## Ethics Statement

The studies involving human participants were reviewed and approved by the studies involving human participants were reviewed and approved by the board at the Faculty of Psychology (Facultad de Psicología) and the General Directorate of Research (Dirección General de Investigaciones) of the Universidad de la Sabana granted ethical approval for the study (Acta CAG #1517 of 19/11/2015). Permission for data collection was granted in agreement with the legal ruling of resolution N° 008430 of 1993 of the Ministerio de Salud de la República de Colombia (Health Ministry of Colombia), which sets out ethical, scientific, technical and administrative norms for research activity with human participants. Written informed consent to participate in this study was provided by the participants’ legal guardian/next of kin.

## Author Contributions

JG-H developed the CARE booklet, supervised the collection and data scoring, performed the statistical analysis, coordinated, and drafted the manuscript. GS participated in the study design and data analytic approach and helped to draft the manuscript. Both authors read and approved the final manuscript.

## Conflict of Interest

The authors declare that the research was conducted in the absence of any commercial or financial relationships that could be construed as a potential conflict of interest.

## Publisher’s Note

All claims expressed in this article are solely those of the authors and do not necessarily represent those of their affiliated organizations, or those of the publisher, the editors and the reviewers. Any product that may be evaluated in this article, or claim that may be made by its manufacturer, is not guaranteed or endorsed by the publisher.

## Footnotes

1
https://monitoreoencasa.weebly.com/
