# Supplementary material for: Agreement and Reliability of Parental Reports and Direct Screening of Developmental Outcomes in Toddlers at Risk
Source: Front Psychol. 2021 Sep 28;12:725146. doi: 10.3389/fpsyg.2021.725146 (PMC8505716; doi:10.3389/fpsyg.2021.725146)
Supplement: Supplementary file 1 [file Table_1.DOCX]

**Supplementary Table 1.** Youden J indexes for empirical values in ROC curve

| True positive rates (sensitivity) | Specificity (sp) | Distance to Sensitivity-Specificity (1,1) point | Youden’s J (YJ) index |
| --- | --- | --- | --- |
| 0.910 | 0.95 | 0.103 | **0.860** |
| 0.931 | 0.90 | 0.121 | **0.831** |
| 0.943 | 0.85 | 0.160 | **0.793** |
| 0.952 | 0.80 | 0.206 | **0.752** |
| 0.958 | 0.75 | 0.253 | **0.708** |
| 0.964 | 0.70 | 0.302 | **0.664** |
| 0.968 | 0.65 | 0.351 | **0.618** |
| 0.972 | 0.60 | 0.401 | **0.572** |
| 0.976 | 0.55 | 0.450 | **0.526** |
| 0.979 | 0.50 | 0.500 | **0.479** |
| 0.982 | 0.45 | 0.550 | **0.432** |
| 0.985 | 0.40 | 0.600 | **0.385** |
| 0.987 | 0.35 | 0.650 | **0.337** |
| 0.989 | 0.30 | 0.700 | **0.289** |
| 0.991 | 0.25 | 0.750 | **0.241** |
| 0.993 | 0.20 | 0.800 | **0.193** |
| 0.995 | 0.15 | 0.850 | **0.145** |
| 0.997 | 0.10 | 0.900 | **0.097** |
| 0.998 | 0.05 | 0.950 | **0.048** |

**Supplementary Table 2.** Personal-social items agreement.

| Item description | *X*^2^ | *p* | *κ* | *Φ* or *Cramer's V* |
| --- | --- | --- | --- | --- |
| 1. Help in House | 19.7 | **< .001** | **.41** | **.80** |
| 1. Feed doll | 32.4 | **< .001** | **.71** | **.62** |
| 1. Remove Garment | 31.1 | **< .001** | **.76** | **.83** |
| 1. When he or she play with dolls, he/she performed a play like a script or short tale with their dolls or toys? | 6.33 | .253 | .01 | .19 |
| 1. Put on clothing | 4.44 | .306 | .03 | .23 |
| 1. Did he/she suggest or show when need to go to the toilet? | 4.91 | **.039** | **-.001** | **.34** |
| 1. Did he/she answer if he or she is a boy or a girl? | 11.2 | **.002** | **.12** | **.44** |
| 1. Dress, no help | 4.23 | .800 | .03 | .19 |
| 1. Did he/she play with an adult using hand puppets? | 3.60 | .541 | .02 | .14 |
| 1. Prepare cereal (In Spanish this item is open to more food than cereals) | 17.9 | **.011** | **.28** | **.34** |
| 1. Draw a person | 17.4 | **.009** | **.03** | **.34** |

Note. Significant *p* values (<.05) in bold.

**Supplementary Table 3.** Language and logico-mathematical reasoning items agreement

| Item description | *X*^2^ | *p* | *κ* | *Φ* or *Cramer's V* |
| --- | --- | --- | --- | --- |
| 1. Name __ Pictures (6 pictures) | 28.9 | **< .001** | **.453** | **.656** |
| 1. Know 2 actions | 28.9 | **< .001** | **.375** | **.728** |
| 1. Combine words | 27.9 | **< .001** | **.376** | **.616** |
| 1. Name __ Pictures (5 pictures) | 44.2 | **< .001** | **.681** | **.742** |
| 1. Use of 3 Objects | 38.6 | **< .001** | **.513** | **.667** |
| 1. Speech half understandable | 24.1 | **< .001** | **.108** | **.571** |
| 1. Did he/she point the dog correctly? (memorize an image) | 8.36 | **.046** | **.236** | **.275** |
| 1. When he or she speaks use pronouns? | 15.0 | **.006** | **.167** | **.356** |
| 1. Did he/she count aloud two consecutive numbers? | 13.8 | **.005** | **.107** | **.406** |
| 1. Name __ Pictures (10 pictures) | 6.53 | **.049** | **.020** | **.501** |
| 1. Did he/she use “to be” in a phrase? | 10.2 | .380 | .048 | .246 |
| 1. Pick longer line | 7.30 | .435 | .006 | .213 |
| 1. Speech all understandable | 11.4 | .329 | .045 | .209 |
| 1. Identify colours | 22.3 | **< .001** | **.272** | **.502** |
| 1. Did he/she realize no-connected actions? | 30.3 | **< .001** | **.299** | **.473** |
| 1. Name colours | 21.4 | **.001** | **.256** | **.453** |
| 1. Opposites - morning/afternoon | 16.4 | .092 | .150 | .370 |
| 1. Did he/she tell stories? | 14.4 | **.008** | **.205** | **.354** |
| 1. Did he/she repeat a complete phrase? | 10.0 | .752 | .062 | .232 |
| 1. Did he/she recognize numbers (Arabic writing numerals)? | 10.3 | .671 | .094 | .232 |

Note. Significant *p* values (<.05) in bold.

**Supplementary Table 4.** Fine motor-adaptive items agreement

| Item description | *X*^2^ | *p* | *κ* | *Φ* or *Cramer's V* |
| --- | --- | --- | --- | --- |
| 1. Put Block in Cup | 11.5 | **.006** | **.212** | **.471** |
| 1. Tower of 4 cubes | 5.60 | **.037** | **.149** | **.328** |
| 1. Thumb-finger grasp (grab a pencil) | 5.26 | .221 | -.009 | .243 |
| 1. Copy a circle | 2.52 | .721 | .015 | .137 |
| 1. Did he/she imitate a bridge with 3 cubes? | 1.15 | .960 | -.030 | .070 |
| 1. Did he/she fold a paper sheet? | 25.4 | **< .001** | **.299** | **.423** |
| 1. Did he/she use scissors to cut a paper sheet? | 18.4 | **.008** | **.196** | **.343** |
| 1. Copy a square | 17.8 | **.002** | **.125** | **.450** |
| 1. Did he/she imitate a door with 5 cubes? | 9.97 | .069 | .169 | .339 |

Note. Significant *p* values (<.05) in bold.

**Supplementary Table 5.** Gross motor items agreement.

| Item description | *X*^2^ | *p* | *κ* | *Φ* or *Cramer's V* |
| --- | --- | --- | --- | --- |
| 1. Walk down steps | 20.7 | **< .001** | **.269** | **.631** |
| 1. Kick ball forward | 14.5 | **< .001** | **.341** | **.631** |
| 1. Broad jump | 7.90 | **.046** | **.091** | **.359** |
| 1. Balance Each Foot 5 seconds | 4.11 | .357 | -.034 | .216 |
| 1. Jump up | 1.33 | 1.000 | .009 | .105 |
| 1. Did he/she jump backwards? | 5.74 | .411 | .038 | .260 |
| 1. Balance each foot 1 second | 11.1 | .215 | .120 | .299 |

Note. Significant *p* values (<.05) in bold.
